# Supplementary material for: Comparing variable and feature selection strategies for prediction - protocol of a simulation study in low-dimensional transplantation data
Source: PLoS One. 2025 Aug 1;20(8):e0328696. doi: 10.1371/journal.pone.0328696 (PMC12316309; doi:10.1371/journal.pone.0328696)
Supplement: S1 — We describe the simulation setup in flowcharts. (PDF) [file pone.0328696.s001.pdf]

Supporting information S1 for: Comparing variable and feature selection strategies for prediction - protocol of a simulation study in low-dimensional transplantation data

Linard Hoessly[1], Jaromil Frossard[1], Simon Schwab[2], Frédérique Chammartin[3], Alexander Leichtle[4], Peter Werner Schreiber[5], Dionysios Neofytos[6], Michael Koller[1], and the Swiss Transplant Cohort Study (STCS)[7]

[1] *Data Center of the Swiss Transplant Cohort Study, University hospital Basel, Basel, Switzerland*
[2] *Swisstransplant, Bern, Switzerland*
[3] *Department of Clincial Research, University Hospital Basel, Basel, Switzerland*
[4] *Cantonal Hospital Baden, Baden, Switzerland*
[5] *Department of Infectious Diseases and Hospital Epidemiology, University Hospital Zurich and University Zurich, Zurich, Switzerland*
[6] *Transplant Infectious Diseases Unit, Service of Infectious Diseases, University Hospitals Geneva, University of Geneva, Geneva, Switzerland*
[7] *Association Swiss Transplant Cohort Study, Switzerland*

July 12, 2025

1 S1: Visualisation of the simulation setup

First we give a simplified overview for how the simulation design is planned, and then a more detailed flowchart.

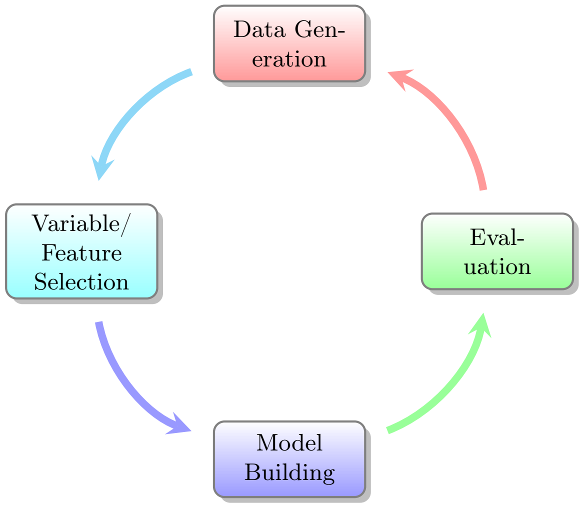

Figure 1: Rough overview.

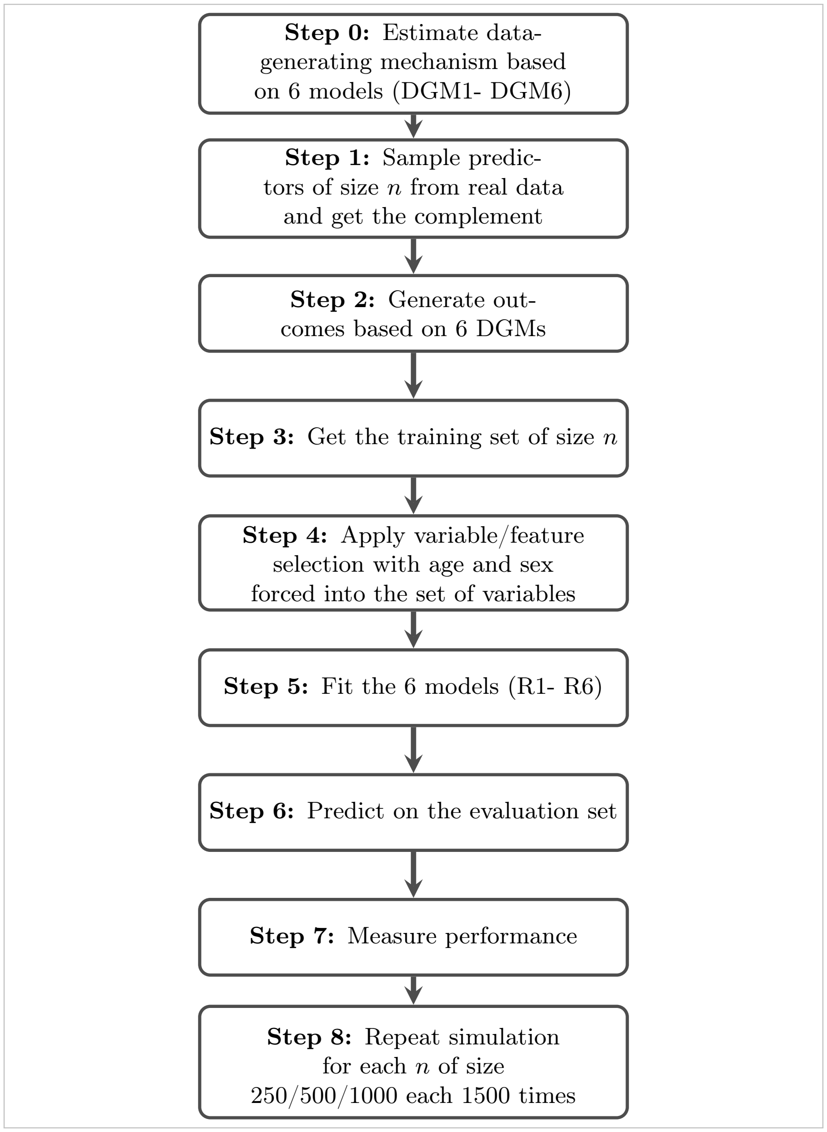

Figure 2: Detailed flow diagram.

Hence, overall, we will have 1500 simulation for each sample sizes of $250, 500, 1000$ observations. In each we will have 6 data generating mechanisms (DGM1-DGM6), 23 variable selection strategies, for 6 regression/ML models (R1-R6), totalling in

$$1500 \cdot 3 \cdot 6 \cdot 23 \cdot 6 = 3726000$$

simulation runs overall.
