# Supplementary material for: Comparing variable and feature selection strategies for prediction - protocol of a simulation study in low-dimensional transplantation data
Source: PLoS One. 2025 Aug 1;20(8):e0328696. doi: 10.1371/journal.pone.0328696 (PMC12316309; doi:10.1371/journal.pone.0328696)
Supplement: S2 — We provide more information on the database and give information on dependent and independent variable with respect to generation of the DGMs 1–6 considered. (PDF) [file pone.0328696.s002.pdf]

Supporting information S2 for: Comparing variable and feature selection strategies for prediction - protocol of a simulation study in low-dimensional transplantation data

Linard Hoessly[1], Jaromil Frossard[1], Simon Schwab[2], Frédérique Chammartin[3], Alexander Leichtle[4], Peter Werner Schreiber[5], Dionysios Neofytos[6], Michael Koller[1], and the Swiss Transplant Cohort Study (STCS)[7]

[1] *Data Center of the Swiss Transplant Cohort Study, University hospital Basel, Basel, Switzerland*
[2] *Swisstransplant, Bern, Switzerland*
[3] *Department of Clincial Research, University Hospital Basel, Basel, Switzerland*
[4] *Cantonal Hospital Baden, Baden, Switzerland*
[5] *Department of Infectious Diseases and Hospital Epidemiology, University Hospital Zurich and University Zurich, Zurich, Switzerland*
[6] *Transplant Infectious Diseases Unit, Service of Infectious Diseases, University Hospitals Geneva, University of Geneva, Geneva, Switzerland*
[7] *Association Swiss Transplant Cohort Study, Switzerland*

July 12, 2025

1 S2: Description of population and variables on which the planned simulation is based

This study is part of a nested project on variable selection for transplant data within the Swiss Transplant Cohort Study (STCS, www.stcs.ch, ClinicalTrials.gov Identifier: NCT01204944). The responsible cantonal Ethics Committee (Ethikkommission Nordwest- und Zentralschweiz, Req. 2023-01812) approved this nested study. The STCS dataset encompasses prospectively collected information on all SOTs performed after 1st May 2008.

1.0.1 Description of the population on which the analysis data is based

3395 adult consenting kidney-transplant recipients registered in the STCS between May 2008 and December 2021 will be included in the study. This data will be used for estimating the independent variable sampling distribution. Complete case analysis will be used for the estimations of the statistical learning/ML models.

1.0.2 Description of the dependent variable

The outcome of experiencing a proven bacterial infection in the 12 months following kidney transplantation is planned to be considered. A proven bacterial infection is defined as detection of a bacterial pathogen with associated clinical signs and symptoms as well as the administration of an appropriate antibiotic treatment [1].

1.0.3 Description of the choice of independent variables

The variables given below in the tables were chosen based on their potential clinical relevance for bacterial infection, and their availability at baseline. We will use some abbreviations in the variable descriptions in the variable names below[1].

[1] Abbreviations: ATG, Anti-Thymocyte Immunogloblin; BAS , Basiliximab; Tac, Tacrolimus; CsA ,Cyclosporin-A; DGF, delayed graft function; tpx, transplantation; cp, cardiopulmonary; cmv, Cytomegalovirus.

- **Categorical**:

Variable	Type of variable	Values	Included in DGM estimation
Induction ATG-containing	categorical	Yes/No	Yes
Induction BAS-containing	categorical	Yes/No	Yes
Maintenance Tac containing	categorical	Yes/No	Yes
Maintenance CsA containing	categorical	Yes/No	Yes
Sex	categorical	M/F	Yes
DGF	categorical	Yes/No	Yes
pre-tpx diabetes	categorical	Yes/No	Yes
pre-tpx hypertension	categorical	Yes/No	Yes
pre-tpx dialysis	categorical	Yes/No	Yes
donor type	categorical	Living vs Deceased	Yes
haldrmismatch	categorical	0 vs 1 vs 2	No
cp problem	categorical	Yes/No	No
cmv risk group	categorical	D+/R- vs D-R- vs R+	Yes
donor sex	categorical	M/F	No

- **Numeric**:

Variable	Type of variable	Unit	Included in DGM estimation
age	numeric	years, precision=2	Yes
cold ischemia time	numeric	min	Yes
BMI	numeric	kg/m^2	Yes
donor age	numeric	years, precision=2	Yes
creatinine	numeric	μmol/l	No

Remark 1.1. Note the following.

- Not all variables were used for the construction of the DGM. Overall, 12 categorical and 4 numerical variables were used in the estimation and construction of the DGM motivated by their potential clinical relation to the outcome of interest.
- The three categorical variables haldrmismatch, cp problem, donor sex, as well as the numerical variable creatinine were not included in the estimation of the DGMs for clinical reasons, such that they act as noise variables in the variable selection and prediction model building part.
- Note that the induction and maintenance variable are determined by the time around transplantation, with induction drugs checked for induction from-7 to +10 days around transplant and maintenance from-1 to +14 days around transplant.

References

[1] van Delden C, Stampf S, Hirsch HH, Manuel O, Meylan P, Cusini A, et al. Burden and Timeline of Infectious Diseases in the First Year After Solid Organ Transplantation in the Swiss Transplant Cohort Study. Clinical Infectious Diseases. 2020;71(7):e159–e169. doi:10.1093/cid/ciz1113.
