# Supplementary material for: Comparing variable and feature selection strategies for prediction - protocol of a simulation study in low-dimensional transplantation data
Source: PLoS One. 2025 Aug 1;20(8):e0328696. doi: 10.1371/journal.pone.0328696 (PMC12316309; doi:10.1371/journal.pone.0328696)
Supplement: Attachment — Submitted filename: response.pdf [file pone.0328696.s003.pdf]

Response to Reviewers

July 2, 2025

Dear Editor,

Please find attached the revised manuscript "Comparing variable and feature selection strategies for prediction - protocol of a simulation study in low-dimensional transplantation data" which we submit for PLOS ONE.

We appreciate the comments and suggestions made by the reviewers and the academic editor(s). We have made corrections in the revised version in blue in the track-changed version. We give point-to-point replies for all issues raised in this rebuttal letter, ordered by main topic.

Sincerely,
Linard Hoessly, on behalf of all authors.

Journal Requirements:

When submitting your revision, we need you to address these additional requirements.

1. Please ensure that your manuscript meets PLOS ONE's style requirements, including those for file naming. The PLOS ONE style templates can be found for main body and authors/affiliation

A: We checked again the format requirement for the main body, added double spacing in latex, and adapted the equations environment for formulas. We checked again the format requirement for authors and affiliations and adapted the corresponding and the group authorship.

2. We note that the grant information you provided in the Funding Information and Financial Disclosure sections do not match. When you resubmit, please ensure that you provide the correct grant numbers for the awards you received for your study in the Funding Information section.

A: To comply with your request we added a section Funding Information after acknowledgements copying the financial disclosure.

3. Thank you for stating the following financial disclosure:
[This project (FUP 214) has been facilitated by the Swiss Transplant Cohort Study. The Swiss Transplant Cohort Study is supported by the Swiss National Science Foundation(SNSF grant 33CS30_201385), Unimedsuisse and the Transplant Centers.].

Please state what role the funders took in the study. If the funders had no role, please state: "The funders had no role in study design, data collection and analysis, decision to publish, or preparation of the manuscript."

If this statement is not correct you must amend it as needed.

Please include this amended Role of Funder statement in your cover letter; we will change the online submission form on your behalf.

A: We included a request to add the sentence "The funders had no role in study design, data collection and analysis, decision to publish, or preparation of the manuscript." in the cover letter.

4. Your ethics statement should only appear in the Methods section of your manuscript. If your ethics statement is written in any section besides the Methods, please move it to the Methods section and delete it from any other section. Please ensure that your ethics statement is included in your manuscript, as the ethics statement entered into the online submission form will not be published alongside your manuscript.

A: We double-checked that the ethics statement is only in the Methods section.

5. One of the noted authors is a group [the Swiss Transplant Cohort Study]. In addition to naming the author group, please list the individual authors and affiliations within this group in the acknowledgments section of your manuscript. Please also indicate clearly a lead author for this group along with a contact email address.

A: We double checked the author group, and provided lead author and contact email.

6. Please review your reference list to ensure that it is complete and correct. If you have cited papers that have been retracted, please include the rationale for doing so in the manuscript text, or remove these references and replace them with relevant current references. Any changes to the reference list should be mentioned in the rebuttal letter that accompanies your revised manuscript. If you need to cite a retracted article, indicate the article?s retracted status in the References list and also include a citation and full reference for the retraction notice.

A: We checked the documentation on formatting references for PLOS ONE, checked for retractions, and removed all URL DOIs.

Reviewer #1:

This paper is acceptable because it presents a clearly structured and well-justified simulation study protocol. The use of the ADEMP framework ensures methodological rigor, the inclusion of both classical and ML methods proves its relevance.

Also, the reliance on real-world transplantation data lends practical credibility. The study?s transparency, thorough documentation, and reproducibility also align well with the standards of a protocol paper.

A:Thank you, we appreciate your comments and critique.

Reviewer #2:

I do not have any major concern.

The protocol is precise and concise.Three suggestions: 1) Provide a stronger rationale for the study. The current transformer-based ML methods have largely de-emphasizes the need for feature selection. Who would be most benefited from this study despite transformer-based, self-supervised models being available? 2) Please elaborate more on preselected variables based on opinions from clinical experts. 3) How will the results be presented for easy and clear comparison? Do the authors have a framework in mind?

A:Thank you, we appreciate your comments and critique.

Concerning (1), we added a sentence on line 13-16 in order to make the motivation more clear. Concerning (2) we added more details on preselection of variables on lines 356-360. For (3) we added a sentence on line 336-340 to give an idea of the framework we have in mind. Since the effectiveness of visualisations depends heavily on the underlying values and the simulations have not yet been run, it is currently not possible to be more specific on the final visual formats.
